# Trends in Characteristics and Treatment of Patients With Chronic Limb-Threatening Ischemia Undergoing Endovascular Therapy

**DOI:** 10.1016/j.jacasi.2025.06.019

**Published:** 2025-08-21

**Authors:** Yosuke Hata, Osamu Iida, Sho Nakao, Taku Toyoshima, Motoki Yasunaga, Hiroaki Nohara, Akito Kawamura, Haruya Yamane, Kuniyasu Ikeoka, Yohei Sotomi, Toshiaki Mano, Yasushi Sakata

**Affiliations:** a Kansai Rosai Hospital Cardiovascular Center, Amagasaki, Hyogo, Japan; b Cardiovascular Division, Osaka Police Hospital, Osaka, Osaka, Japan; c Division of Cardiology, Osaka Rosai Hospital, Sakai, Osaka, Japan; d Cardiovascular Division, NHO Osaka National Hospital, Osaka, Osaka, Japan; e Department of Cardiovascular Medicine, Osaka University Graduate School of Medicine, Suita, Osaka, Japan

**Keywords:** chronic limb-threatening ischemia, endovascular therapy, major amputation, mortality, treatment trend

## Abstract

**Background:**

Due to an aging population and the growing prevalence of diabetes, patients with chronic limb-threatening ischemia (CLTI) represent a growing burden on health care and social services.

**Objectives:**

This study aimed to investigate the trends in characteristics and treatment of patients with CLTI.

**Methods:**

This multicenter retrospective study included 2,085 patients with CLTI who underwent endovascular therapy between April 2010 and March 2023. Clinical characteristics and outcomes were compared among 4 groups based on treatment period quartiles (Q1-Q4). The clinical characteristics of the patients, limbs, and anatomical severity were assessed according to the global vascular guidelines. The outcome measures were mortality or major amputation rate, and reintervention rate.

**Results:**

The median follow-up period was 14.9 months (IQR: 3.9-36.5 months). In the later periods, patient risk was higher, wound severity was lower, and anatomical severity was higher in inframalleolar lesions (each *P* < 0.05). The mortality or major amputation rate at 1 year did not significantly differ across Q1-Q4 (24.2% [95% CI: 20.5%-28.5%], 22.7% [95% CI: 19.0%-27.0%], 25.6% [95% CI: 21.8%-30.0%], and 28.9% [95% CI: 24.7%-33.5%] in Q1-Q4, respectively; *P* for trend = 0.18). The reintervention rate at 1 year was significantly lower in the later periods (43.2% [95% CI: 38.5%-48.3%], 45.2% [95% CI: 40.4%-50.4%], 37.8% [95% CI: 33.2%-42.8%], and 32.5% [95% CI: 27.9%-37.6%] in Q1-Q4, respectively; *P* for trend = 0.002).

**Conclusions:**

The characteristics of patients with CLTI were significantly different across the treatment period. The mortality or major amputation rates did not significantly different, whereas the reintervention rate was significantly lower in the later periods.

The aging population and diabetes epidemic have led to a global increase in the prevalence of lower-extremity arterial disease, including chronic limb-threatening ischemia (CLTI), which is the most severe manifestation of lower-extremity arterial disease. Without appropriate treatment, these conditions are associated with poor limb and life prognosis.^1^^,^^2^

Regarding the clinical outcomes of endovascular therapy (EVT) vs surgical bypass, two randomized controlled trials, the BEST-CLI (Best Endovascular versus Best Surgical Therapy in Patients with Critical Limb-Threatening Ischemia) and BASIL-2 (Bypass versus Angioplasty in Severe Ischaemia of the Leg-2),^3^^,^^4^ have demonstrated conflicting results. However, due to the accumulation of technical experience and the development of endovascular devices, a less invasive endovascular approach has gained increasing acceptance among real-world patients with CLTI and complex comorbidities compared with surgical interventions.^5^ Several real-world observational studies have also offered valuable insights into clinical outcome of CLTI undergoing EVT. The SPINACH (Surgical Reconstruction Versus Peripheral Intervention in Patients with Critical Limb Ischemia) study compared endovascular and surgical revascularization over 3 years, finding comparable outcomes.^6^ Meanwhile, CRITISCH (the Registry of First-Line Treatments in Patients With Critical Limb Ischemia) showed that an endovascular-first strategy was not inferior to surgical bypass.^7^ Nevertheless, each registry was limited by a specific enrollment period, making it difficult to clarify whether the clinical characteristics and outcomes of patients with CLTI undergoing EVT have changed across the era. Therefore, this study aimed to investigate the trends in characteristics and treatment of patients with CLTI undergoing EVT.

## Methods

### Study participants

This retrospective, multicenter study used the SAPLING (SAtellite database of Patients with chronic LImb-threateniNG ischemia), consisting of 2,162 patients with CLTI who underwent EVT between April 2010 and March 2023 at participating centers. We excluded 77 patients with missing data, and the remaining 2,085 patients were included in the present study. The clinical characteristics and outcomes were compared among 4 groups classified based on the quartiles of the treatment periods (Q1-Q4); first (April 1, 2010 to March 24, 2014), second (March 25, 2014 to September 30, 2017), third (October 1, 2017 to July 14, 2020), and fourth quarters (July 15, 2020 to March 31, 2023). We collected data from electronic medical records at 4 cardiovascular centers located in Osaka and Hyogo Prefectures, Japan. We identified patients diagnosed with CLTI who underwent EVT and extracted relevant clinical, anatomical, and outcome variables from the electronic medical records. The study was performed in accordance with the Declaration of Helsinki and approved by the ethics committee of each participating hospital. The current analysis involved observational research without intervention or invasiveness and did not use human biological specimens. Therefore, the requirement for written informed consent from patients was waived following the Ethical Guidelines for Medical and Health Research Involving Human Subjects in Japan. Alternatively, relevant study information was made available to the public and opportunities for individuals to refuse the inclusion of their data were ensured. This report followed the STROBE guidelines for observational studies and the STROBE checklist was provided as Supplemental Table 1.

### Baseline assessment and revascularization strategy

The severity of ischemia in the index limb was routinely evaluated using the ankle-brachial index and skin perfusion pressure (SPP). The severity and location of the lower limb arterial lesions were evaluated using duplex ultrasound scanning, and the presence of significant arterial lesions was verified using angiography before revascularization. Revascularization was indicated for the target lesions with angiographic stenosis of ≥75% of the vessel diameter, and hemodynamically significant evidence as pressure gradient of ≥10 mm Hg was presented. The revascularization strategy depended on the physician’s discretion. Generally, a primary stenting strategy is used for aortoiliac lesions, and a provisional stenting strategy is used for femoropopliteal (FP) lesions. Infrapopliteal (IP) lesions were treated with plain angioplasty. Atherectomy devices were not approved for use in Japan during the study period.

### Follow-up assessment and management

Follow-up intervals and modalities were determined by attending physician. Patients were followed every 2 to 4 weeks until the wound healed. Reinterventions were indicated in cases of recurrent ischemic pain or delayed wound healing, accompanied by recurrent occlusion or stenosis, and hemodynamic indications based on the ankle-brachial index, deep ultrasound scanning, and SPP.

### Definitions

CLTI was defined according to the Global Vascular Guidelines (GVG).^2^ Any amputation at or distal to the Lisfranc ligament was not considered a limb salvage failure. Nonambulatory status was defined as wheelchair dependence or bedridden status, as assessed upon admission. The Wound, Ischemia, and foot Infection (WIfI)^8^ classification was used to estimate the 1-year amputation risk, which was retrospectively determined using photographs of the pedal wounds and medical records, including laboratory examinations on admission. Preprocedural angiography was used to assess the GLASS (Global Limb Anatomic Staging System).^2^ High-risk patients were defined as having estimated perioperative and 2-year mortality rates >5% and >50%, respectively, based on the GVG. The mortality rates were estimated using the SPINACH score.^9^ We also calculated according to the PREVENT III (Project of Ex-Vivo graft Engineering via Transfection III) score to predict 1-year amputation-free survival rate.^10^ Balloon- and stent-based treatment were defined as treatment with plain balloons and bare metal stents, respectively. Antirestenotic treatment was defined as treatment with drug-coated balloons, drug-eluting stents, or stent grafts. Arterial calcification was assessed using a PACSS (Peripheral Arterial Calcification Scoring System).^11^

### Outcome measures

The outcome measures were the mortality or major amputation rate and reintervention rate.

### Statistical analysis

Data were presented as numbers (percentages) and median (IQR) for continuous and discrete variables unless otherwise specified. Nonparametric analysis for continuous variables, chi-square test for discrete variables, and Mann-Whitney *U* test for ordinal variables were performed to assess differences in baseline characteristics between the groups. We applied the Cochran-Armitage trend test to analyze trends in the patient, limb, and anatomical risk scoring systems. The mortality or major amputation rate and reintervention rate were estimated using the Kaplan-Meier method, and differences between groups were evaluated using log-rank and log-rank trend tests when necessary. The association between reintervention and baseline characteristics was evaluated using a Cox regression hazard model with HRs and 95% CIs. Following clinical variables found to influence reintervention in previous studies^12^, ^13^, ^14^ and selected based on our clinical consensus were included in multivariate analysis: sex, age, non-ambulatory status, tissue loss, diabetes mellitus, hemodialysis, chronic total occlusion, lesion length, PACSS grade, GLASS stage, number of IP runoff vessels, and antirestenotic treatment. The proportional hazards assumption was tested with Schoenfeld residuals (global *P* = 0.13; no variable violated the assumption). Additionally, as a sensitivity analysis for reintervention, we performed a Fine-Gray test treating death or major amputation as competing events. We evaluated the follow-up index of each patient group to variate to avoid attrition bias.^15^ The independent associations were explored using multivariate models. In cases where both limbs were treated, the limb with the higher WIfI stage was used for data analysis to capture the more severe presentation. Regarding to missing data, baseline characteristics of cases with and without missing data are presented in Supplemental Table 2. Additionally, a multivariate logistic regression model showed that hemodialysis, tissue loss, and GLASS stage were associated with data missingness (Supplemental Table 3) (all *P* < 0.05), indicating data were not missing completely at random but plausibly missing at random. These predictors, together with all outcome-related variables, were included in the imputation model to satisfy the missing at random condition in multiple imputation by the chained equations method. Missing data were addressed using multiple imputation. During the procedure, five imputed datasets were generated and the analytic results were combined according to Rubin’s rule.^16^ Statistical significance was set at *P* < 0.05. Statistical analyses were performed using R software version 4.3.1 (The R Foundation for Statistical Computing).

## Results

The median follow-up period was 14.9 months (IQR: 3.9-36.5 months). The baseline characteristics of the patients are demonstrated in Table 1. In the later periods among Q1-Q4, more older patients (median 74 [IQR: 68-81] years, 75 [68-82] years, 76 [69-83] years, and 78 [71-84] years in Q1-Q4, respectively), and more patients with heart failure (2.7% [14/521], 9.6% [50/521], 18.0% [94/522], and 17.3% [90/521] in Q1-Q4, respectively), higher preoperative SPP levels (median 21 [IQR: 14-33] mm Hg, 25 [IQR: 17-36] mm Hg, 25 [IQR: 16-37] mm Hg, and 29 [IQR: 20-42] mm Hg in Q1-Q4, respectively), and lower serum albumin levels (median 3.4 [IQR: 3.0-3.8] g/dL, 3.3 [IQR: 2.8-3.7] g/dL, 3.3 [IQR: 2.8-3.7] g/dL, and 29 [IQR: 20-42] g/dL in Q1-Q4, respectively) were observed. Regarding patient risk, the later periods saw more high-risk patients based on the SPINACH scores (*P* = 0.006; 20.5% [107/521], 15.9% [83/521], 24.5% [128/522], and 25.7% [134/521] in Q1-Q4, respectively) but not based on the PREVENT III score (*P* = 0.081; 42.0% [219/521], 40.5% [211/521], 46.7% [244/522], and 45.7% [238/521] in Q1-Q4, respectively). Limb severity, assessed as high risk according to the WIfI classification, was more common in the earlier periods (*P* < 0.001; 47.6% [247/521], 35.9% [187/521], 34.4% [179/522], and 35.5% [184/521] in Q1-Q4, respectively), and anatomical severity with GLASS inframalleolar (IM) P1 lesions was more frequent in the later periods, with significant differences (*P* = 0.017; 24.8% [129/521], 38.8% [202/521], 37.0% [193/522], and 45.5% [237/521] in Q1-Q4, respectively). Furthermore, there was a trend toward a decrease in CLTI patients preferred for surgical bypass by the GVG, defined as those with GLASS stage 3, WIfI stage 3 or 4, and average surgical risk, in the later periods (*P* = 0.004; 35.1% [183/521], 33.6% [175/521], 30.7% [160/522], and 28.8% [150/521] in Q1-Q4, respectively).

**Table 1:** Baseline Characteristics of Patients

	Q1 (n = 521)	Q2 (n = 521)	Q3 (n = 522)	Q4 (n = 521)	*P* Value
Patient characteristics					
Male	311 (59.7)	324 (62.2)	308 (59.0)	328 (63.0)	0.50
Age, y	74 (68-81)	75 (68-82)	76 (69-83)	78 (71-84)	<0.001
Body mass index, kg/m^2^	21.1 (18.8-23.5)	21.0 (18.5-23.4)	21.5 (19.2-24.1)	20.9 (18.6-23.6)	0.072
Non-ambulatory status	250 (48.0)	220 (42.2)	216 (41.4)	228 (43.8)	0.14
Hypertension	343 (65.8)	320 (61.4)	371 (71.1)	373 (71.6)	0.001
Diabetes mellitus	339 (65.1)	307 (58.9)	329 (63.0)	307 (58.9)	0.10
Dyslipidemia	172 (33.0)	156 (29.9)	294 (56.3)	261 (50.1)	<0.001
Current smoking	171 (32.8)	56 (10.7)	68 (13.1)	74 (14.2)	<0.001
Hemodialysis	254 (48.8)	287 (55.1)	239 (45.8)	229 (44.0)	0.002
Coronary artery disease	214 (41.4)	268 (51.4)	303 (58.0)	265 (50.9)	<0.001
Congestive heart failure	14 (2.7)	50 (9.6)	94 (18.0)	90 (17.3)	<0.001
Left ventricular ejection fraction, %	64 (56-71)	65 (56-69)	64 (55-70)	62 (53-69)	0.02
Serum albumin, g/dL	3.4 (3.0-3.8)	3.3 (2.8-3.7)	3.3 (2.9-3.7)	3.3 (2.8-3.7)	0.003
Medications					
Antiplatelet agent	453 (86.9)	415 (79.7)	482 (92.3)	482 (92.5)	<0.001
Warfarin	114 (21.9)	99 (19.0)	66 (12.6)	32 (6.1)	<0.001
DOAC	4 (0.8)	19 (3.6)	51 (9.8)	86 (16.5)	<0.001
Statin	111 (21.3)	135 (25.9)	236 (45.2)	225 (43.2)	<0.001
ACE inhibitor/ARB	165 (31.7)	196 (37.6)	208 (39.8)	185 (35.5)	0.042
Beta-blocker	123 (23.6)	159 (30.5)	132 (25.3)	94 (18.0)	<0.001
GDMT	148 (28.4)	172 (33.0)	181 (34.8)	168 (32.2)	0.16
Patient risk					
PREVENT III score					0.081
Low risk	87 (16.7)	83 (15.9)	72 (13.8)	73 (14.0)	
Medium risk	211 (40.5)	227 (43.6)	206 (39.5)	210 (40.3)	
High risk	219 (42.0)	211 (40.5)	244 (46.7)	238 (45.7)	
SPINACH score					0.006
Average risk	393 (75.4)	427 (82.0)	393 (75.3)	380 (72.9)	
High risk	107 (20.5)	83 (15.9)	128 (24.5)	134 (25.7)	
Missing data	21 (4.1)	11 (2.1)	1 (0.2)	7 (1.4)	
Limb characteristics					
Ankle-brachial index	0.64 (0.51-0.77)	0.64 (0.50-0.76)	0.62 (0.47-0.77)	0.65 (0.47-0.80)	0.35
Skin perfusion pressure, mm Hg	21 (14-33)	25 (17-36)	25 (16-37)	29 (20-42)	<0.001
Rutherford classification					0.001
4, only rest pain	69 (13.2)	79 (15.2)	87 (16.7)	51 (9.8)	
5, minor tissue loss	339 (65.1)	311 (59.7)	313 (60.0)	370 (71.0)	
6, major tissue loss	113 (21.7)	131 (25.1)	122 (23.4)	100 (19.2)	
Wound grade according to WIfI classification					<0.001
0	69 (13.2)	79 (15.2)	87 (16.7)	51 (9.8)	
1	185 (35.5)	226 (43.4)	239 (45.8)	233 (44.7)	
2	142 (27.3)	118 (22.6)	120 (23.0)	177 (34.0)	
3	104 (20.0)	93 (17.9)	76 (14.6)	59 (11.3)	
Missing data	21 (4.0)	5 (1.0)	0 (0)	1 (0.2)	
Ischemia grade according to WIfI classification					<0.001
1	139 (26.7)	155 (29.7)	121 (23.1)	157 (30.1)	
2	110 (21.1)	136 (26.1)	116 (22.2)	130 (25.0)	
3	257 (49.3)	214 (41.1)	283 (54.2)	225 (43.2)	
Missing data	15 (2.9)	16 (3.1)	2 (0.4)	9 (1.7)	
Foot infection grade according to WIfI classification					<0.001
0	195 (37.4)	245 (47.0)	280 (53.6)	260 (49.9)	
1	103 (19.8)	124 (23.8)	116 (22.2)	143 (27.4)	
2	184 (35.3)	107 (20.5)	106 (20.3)	98 (18.8)	
3	17 (3.3)	40 (7.7)	20 (3.8)	19 (3.6)	
Missing data	22 (4.2)	5 (1.0)	0 (0)	1 (0.2)	
WIfI clinical stage					<0.001
Stage 1, very low risk	56 (10.8)	62 (11.9)	48 (9.2)	64 (12.3)	
Stage 2, low risk	63 (12.1)	128 (24.6)	132 (25.4)	105 (20.2)	
Stage 3, moderate risk	124 (23.9)	128 (24.6)	159 (30.6)	157 (30.3)	
Stage 4, high risk	247 (47.6)	187 (35.9)	179 (34.4)	184 (35.5)	
Missing data	29 (5.6)	16 (3.1)	2 (0.4)	9 (1.7)	
Arterial lesion characteristic					
GLASS FP grade					<0.001
0	198 (38.0)	156 (29.9)	165 (31.6)	171 (32.8)	
1	48 (9.2)	67 (12.9)	74 (14.2)	69 (13.2)	
2	54 (10.4)	80 (15.4)	56 (10.7)	71 (13.6)	
3	64 (12.3)	68 (13.1)	83 (15.9)	74 (14.2)	
4	112 (21.5)	140 (26.9)	128 (24.5)	128 (24.6)	
Missing data	45 (8.6)	10 (1.9)	16 (3.1)	8 (1.5)	
GLASS IP grade					<0.001
0	23 (4.4)	28 (5.4)	53 (10.2)	51 (9.8)	
1	46 (8.8)	37 (7.1)	31 (5.9)	37 (7.1)	
2	61 (11.7)	58 (11.1)	69 (13.2)	69 (13.2)	
3	55 (10.6)	64 (12.3)	89 (17.0)	107 (20.5)	
4	279 (53.6)	302 (58.0)	263 (50.4)	238 (45.7)	
Missing data	57 (10.9)	32 (6.1)	17 (3.3)	19 (3.6)	
GLASS IM grade					0.017
P0	170 (32.6)	131 (25.1)	122 (23.4)	109 (20.9)	
P1	129 (24.8)	202 (38.8)	193 (37.0)	237 (45.5)	
P2	136 (26.1)	106 (20.3)	142 (27.2)	120 (23.0)	
Missing data	86 (16.5)	82 (15.7)	64 (12.3)	55 (10.6)	
GLASS stage					0.43
Stage I	60 (11.5)	60 (11.5)	50 (9.6)	59 (11.3)	
Stage II	72 (13.8)	71 (13.6)	94 (18.0)	96 (18.4)	
Stage III	336 (64.5)	373 (71.6)	350 (67.0)	345 (66.2)	
Missing data	47 (9.0)	14 (2.7)	17 (3.3)	13 (2.5)	
Lesion distribution					
FP	278 (58.4)	355 (69.5)	341 (67.4)	342 (66.7)	0.002
IP	441 (95.0)	461 (94.3)	452 (89.5)	451 (89.8)	0.001
IM	265 (60.9)	308 (70.2)	335 (73.3)	357 (76.6)	<0.001
Treatment modality					<0.001
Ballon-based treatment	337 (64.7)	363 (69.7)	308 (59.0)	272 (52.2)	
Stent-based treatment	134 (25.7)	127 (24.4)	55 (10.5)	16 (3.1)	
Antirestenotic treatment	50 (9.6)	31 (6.0)	159 (30.5)	233 (44.7)	
Preferred revascularization strategy by the GVG					0.004
EVT preferred or indeterminate	259 (49.7)	309 (59.3)	329 (63.0)	334 (64.1)	
BSX preferred	183 (35.1)	175 (33.6)	160 (30.7)	150 (28.8)	
Missing data	79 (15.2)	37 (7.1)	33 (6.3)	37 (7.1)	
Follow-up index	1.0 (1.0-1.0)	1.0 (1.0-1.0)	1.0 (1.0-1.0)	1.0 (1.0-1.0)	0.24

Values are as n (%) or median (IQR).

WIfI clinical stage was used to predict the 1-year amputation risk. Balloon- and stent-based treatments were defined as those using plain balloons and bare metal stents, respectively. Antirestenotic treatment was defined as treatment with drug-coated balloons, drug-eluting stents, or stent grafts. In cases where both limbs were treated, the limb with the higher WIfI stage was used for data analysis to capture the more severe presentation.

ACE = angiotensin-converting enzyme; ARB = angiotensin receptor blocker; BSX = bypass surgery; DOAC = direct oral anticoagulant; EVT = endovascular therapy; FP = femoropopliteal; GDMT = guideline-directed medical therapy (aggregate prescription of antiplatelet agents, statins, and ACE inhibitors or ARBs); GLASS = Global Limb Anatomical Staging System; GVG = Global Vascular Guidelines; IM = inframalleolar; IP = infrapopliteal; Q1–Q4 = first to fourth quartiles; WIfI = Wound, Ischemia, and foot Infections.

Antirestenotic treatment was administered to 9.6% (50/521), 6.0% (31/521), 30.5% (159/522), and 44.7% (233/521) of patients in Q1, Q2, Q3, and Q4, respectively. Details of the treatment modalities are illustrated in Supplemental Table 4.

**Figure 1:**
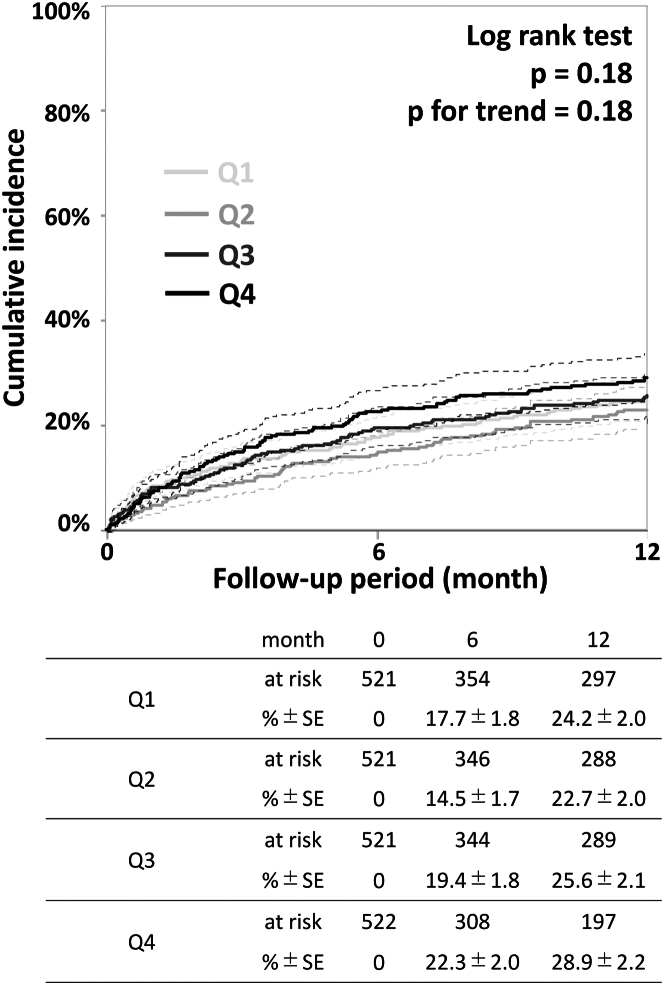
Mortality or Major Amputation Rates One-year mortality or major amputation rates were 24.2% (95% CI: 20.5%-28.5%), 22.7% (95% CI: 19.0%-27.0%), 25.6% (95% CI: 21.8%-30.0%), and 28.9% (95% CI: 24.7%-33.5%) in Q1, Q2, Q3, and Q4, respectively, without significant differences (log-rank *P* and *P* for trend = 0.18). Dashed lines indicate the 95% CIs. Q1–Q4: first to fourth quartiles.

As demonstrated in Figure 1, the 1-year mortality or major amputation rates were 24.2% (95% CI: 20.5%-28.5%), 22.7% (95% CI: 19.0%-27.0%), 25.6% (95% CI: 21.8%-30.0%), and 28.9% (95% CI: 24.7%-33.5%) in Q1, Q2, Q3, and Q4, respectively, with no significant differences (log-rank *P* and *P* for trend = 0.18). Supplemental Figures 1 and 2 demonstrate that both of 1-year mortality and major amputation rates were not significantly different.

**Figure 2:**
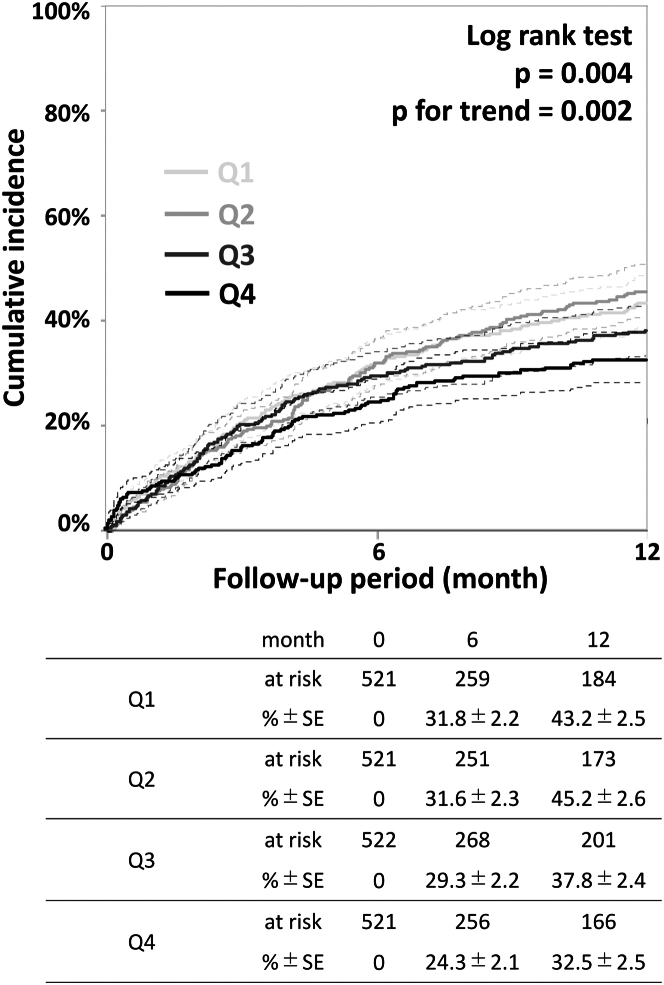
Reintervention Rates One-year reintervention rates were 43.2% (95% CI: 38.5%-48.3%), 45.2% (95% CI: 40.4%-50.4%), 37.8% (95% CI: 33.2%-42.8%), and 32.5% (95% CI: 27.9%-37.6%) in Q1, Q2, Q3, and Q4, respectively, with significant differences (log-rank *P* = 0.004 and *P* for trend = 0.002). Dashed lines indicate the 95% CIs. Q1–Q4: first to fourth quartiles.

**Figure 3:**
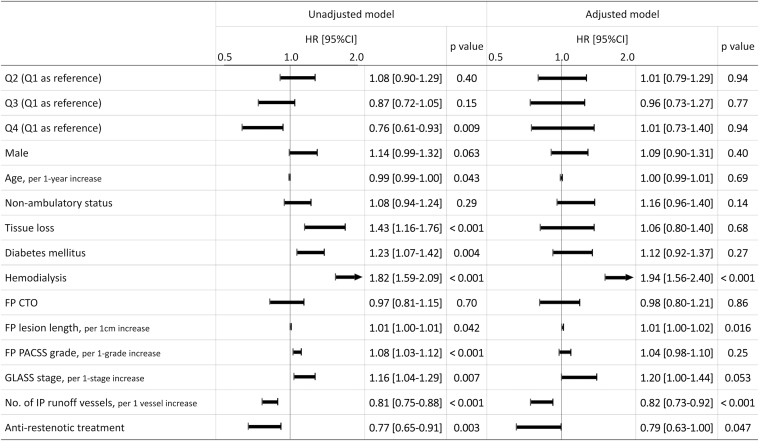
Association Between Baseline Characteristics and Reintervention HRs are presented with 95% CIs. Antirestenotic treatment was defined as treatment with drug-coated balloons, drug-eluting stents, or stent grafts. CTO = chronic total occlusion; FP = femoropopliteal; GLASS = Global Limb Anatomical Staging System; IP = infrapopliteal; PACSS = Peripheral Artery Calcification Scoring System; Q1–Q4 = first to fourth quartiles.

As demonstrated in Figure 2, the 1-year reintervention rates were 43.2% (95% CI: 38.5%-48.3%), 45.2% (95% CI: 40.4%-50.4%), 37.8% (95% CI: 33.2%-42.8%), and 32.5% (95% CI: 27.9%-37.6%) in Q1, Q2, Q3, and Q4, respectively, with significant differences (log-rank *P* = 0.004 and *P* for trend = 0.002). In addition, multivariate analysis revealed that hemodialysis (HR: 1.94 [95% CI: 1.56-2.40]; *P* < 0.001), FP lesion length (per 1-cm increase, HR: 1.01 [95% CI: 1.00-1.02]; *P* = 0.016), number of IP runoff vessels (per 1-vessel increase, HR: 0.82 [95% CI: 0.73-0.92]; *P* < 0.001), and use of antirestenotic treatment (HR: 0.79 [95% CI: 0.63-1.00]; *P* = 0.047) were significantly associated with reintervention, as shown in Figure 3. Additionally, as a sensitivity analysis, a multivariate Fine-Gray test treating death or major amputation as competing events demonstrated consistent result that use of antirestenotic treatment was significantly associated with reintervention (HR: 0.80 [95% CI: 0.73-0.87]; *P* = 0.028]).

## Discussion

### Summary of this study

This study revealed that, over time, patient risk increased, wound severity decreased, and the anatomical severity of IM lesions increased. Additionally, the mortality or major amputation rates did not significantly different across Q1-Q4, whereas the reintervention rates decreased (Central Illustration).

**Central Illustration:**
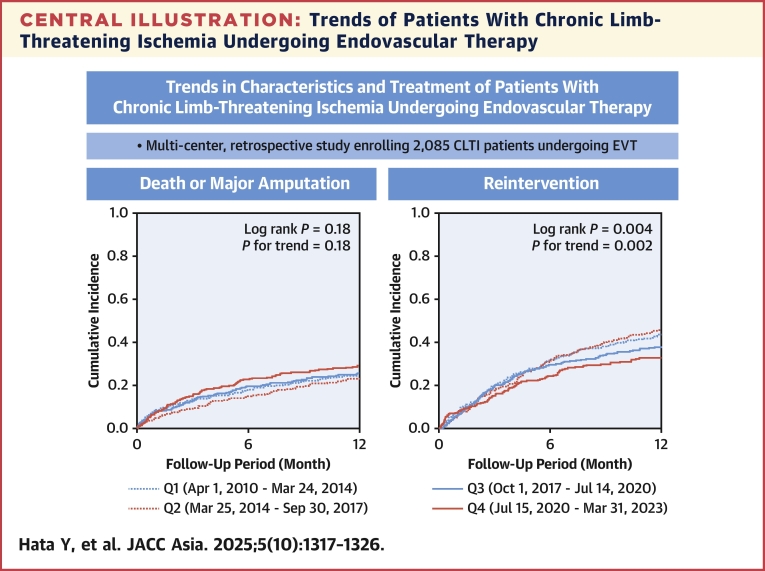
Trends of Patients With Chronic Limb-Threatening Ischemia Undergoing Endovascular Therapy The mortality or major amputation rates did not significantly different across the first to fourth quartiles (Q1–Q4), whereas the reintervention rates decreased. CLTI = chronic limb-threatening ischemia; EVT = endovascular therapy.

### Differences in clinical background

In the later periods, the patients were older, suggesting an aging population and increased complexity of disease profiles. Limb severity, as assessed by the clinical stage in the WIfI classification, was milder in the later periods, which may indicate that the increased awareness of CLTI in the Japanese medical community probably allowed for earlier referral and intervention before the wound condition became severe. The increase in patient risk along with the decrease in wound severity may be attributed to the trend of EVT becoming more mainstream, compared with surgical bypass.

Although no difference existed between the treatment period quartiles in terms of the GLASS stage, GLASS IM P1 lesions were significantly more frequent in the later periods. This may be influenced by the aging of patients with CLTI and an increase in number of long-term dialysis patients, leading to more severe IM lesions.^17^ The severity of IM lesions is a predictor of poor prognosis after EVT and bypass surgery.^18^^,^^19^ Moreover, substantial risk for angiographic deterioration exists after EVT for IM lesions, leading to an increased risk of major amputation.^20^ The findings of this study suggest that the number of patients with challenging CLTI will continue to increase in the future.

### Mortality or major amputation rates

Several studies from national databases in Europe and the United States have revealed a year-by-year decrease in the mortality or major amputation rates among all populations with CLTI, regardless of whether they undergo revascularization.^21^, ^22^, ^23^ The findings differed from those of the present study. The reason for this discrepancy is that, in previous reports, approximately one-half of the patients did not undergo revascularization, whereas in this study, all patients received revascularization.

In the present study, the mortality and major amputation rates did not significantly change during the study period, indicating that the prognosis for patients has not improved. The positive impact of decreased wound severity, due to increased awareness and early intervention, may be offset by the negative impact of age-related comorbidities and more severe anatomical features.

Notably, the arterial lesion characteristics of recent patients with CLTI have shifted toward more distal segments. This suggests that surgical bypass faces challenges in selecting distal anastomosis sites, and EVT encounters cases in which adequate blood flow cannot be achieved due to the lack of suitable outflow vessels.^18^^,^^24^ This trend may become more pronounced in the future. Therefore, comprehensive therapies, including wound care and adjunctive therapies to achieve wound healing with limited blood flow, are becoming increasingly important in CLTI management, rather than revascularization alone.

### Decrease in reintervention rates

In this study, the reintervention rate decreased in the later periods. This may be related to advancements in treatment approaches, such as the development of more durable devices. In FP lesions, contemporary devices represented by drug-coated balloons, drug-eluting stents, and stent grafts have demonstrated better primary patency rates compared with traditional bare-metal stents and plain old balloon angioplasty.^25^, ^26^, ^27^ Furthermore, in the multivariate analysis of this study, antirestenotic treatment was identified as a factor in reducing the risk of reintervention. This finding suggests that maintaining long-term blood flow in the FP lesion may have contributed to the decrease in the reintervention rates. Moreover, the increased application of antirestenotic treatment would be accompanied by a more proactive approach to vessel preparation prior to device deployment, more refined patient selection using the GLASS and WIfI classifications, and field-wide improvements—including standardized wound care, off-loading protocols, and adjunctive therapy.^2^^,^^8^^,^^28^^,^^29^ These comprehensive advances may also have contributed to the observed decline in reintervention rates, and further investigation will be needed to evaluate their individual effects in future studies.

On the other hand, the “Achilles’ heel” in CLTI treatment is IP lesions. In our study, only plain old balloon angioplasty, which has a high recurrence rate, was used to treat IP lesions throughout the study period. Although a recent report has discussed the potential of bioresorbable scaffolds for IP lesions,^30^ it remains uncertain whether game-changing treatments for IP lesions will emerge.

### Clinical implications

These findings of our study suggest that the adoption of antirestenotic devices provides a clear benefit by reducing reintervention rates among patients with CLTI. However, despite these device-related advances, mortality and major amputation rates have not significantly improved in recent years. To achieve better outcomes, a more comprehensive approach would be required—encompasses early screening and referral, guideline-directed medical therapy, optimized wound care, and dedicated foot-care strategies, embedded within coordinated community-based care.

### Study limitations

First, this was an observational study, including potential outcome ascertainment bias due to reliance on electronic medical records, and the limited generalizability of our results stemming from 4 centers in a specific geographic region in Japan. In addition, data from patients who sought follow-up care elsewhere could not be captured. Second, due to missing data inherent in this retrospective study, we were unable to comprehensively assess all confounders. Third, because the registry included only patients who underwent EVT as initial treatment, and those who underwent surgical revascularization, primary amputation, or conservative management were not captured. This limits external comparison across treatment strategies. Finally, data on the reintervention sites were not available.

## Conclusions

This study revealed significant differences in the characteristics of patients with CLTI over time. The mortality or major amputation rates did not significantly differ across the study period, whereas reintervention rates were significantly lower in the later periods.

### Data Statement

The data underlying this article cannot be shared publicly due to ethical reasons, that is, for the privacy of individuals that participated in the study. The data will be shared on reasonable request to the corresponding author and with permission of the ethics committee of the participating institutions.

## Funding Support and Author Disclosures

The authors have reported that they have no relationships relevant to the contents of this paper to disclose.

## References

[1] Song P., Rudan D., Zhu Y. (2019). Global, regional, and national prevalence and risk factors for peripheral artery disease in 2015: an updated systematic review and analysis. Lancet Glob Health.

[2] Conte M.S., Bradbury A.W., Kolh P. (2019). Global vascular guidelines on the management of chronic limb-threatening ischemia. J Vasc Surg.

[3] Farber A., Menard M.T., Conte M.S. (2022). Surgery or endovascular therapy for chronic limb-threatening ischemia. N Engl J Med.

[4] Bradbury A.W., Moakes C.A., Popplewell M. (2023). A vein bypass first versus a best endovascular treatment first revascularisation strategy for patients with chronic limb threatening ischaemia who required an infra-popliteal, with or without an additional more proximal infra-inguinal revascularisation procedure to restore limb perfusion (BASIL-2): an open-label, randomised, multicentre, phase 3 trial. Lancet.

[5] Hata Y., Iida O., Okamoto S. (2024). Japanese real-world population with chronic limb-threatening ischemia who meet the criteria of the BEST-CLI trial. Vasc Med.

[6] Iida O., Takahara M., Soga Y. (2017). Three-year outcomes of surgical versus endovascular revascularization for critical limb ischemia: the SPINACH study (Surgical Reconstruction Versus Peripheral Intervention in Patients With Critical Limb Ischemia). Circ Cardiovasc Interv.

[7] Bisdas T., Borowski M., Stavroulakis K., Torsello G., CRITISCH Collaborators (2016). Endovascular therapy versus bypass surgery as first-line treatment strategies for critical limb ischemia: results of the interim analysis of the CRITISCH registry. JACC Cardiovasc Interv.

[8] Mills J.L., Conte M.S., Armstrong D.G. (2014). The Society for Vascular Surgery Lower Extremity Threatened Limb Classification System: risk stratification based on wound, ischemia, and foot infection (WIfI). J Vasc Surg.

[9] Azuma N., Takahara M., Kodama A. (2019). Predictive model for mortality risk including the wound, ischemia, foot infection classification in patients undergoing revascularization for critical limb ischemia. Circ Cardiovasc Interv.

[10] Schanzer A., Mega J., Meadows J. (2008). Risk stratification in critical limb ischemia: derivation and validation of a model to predict amputation-free survival using multicenter surgical outcomes data. J Vasc Surg.

[11] Rocha-Singh K.J., Zeller T., Jaff M.R. (2014). Peripheral arterial calcification: prevalence, mechanism, detection, and clinical implications. Catheter Cardiovasc Interv.

[12] Iida O., Takahara M., Soga Y. (2014). Shared and differential factors influencing restenosis following endovascular therapy between TASC (Trans-Atlantic Inter-Society Consensus) II class A to C and D lesions in the femoropopliteal artery. JACC Cardiovasc Interv.

[13] Iida O., Takahara M., Soga Y., CAPSICUM Investigators (2022). 1-Year outcomes of fluoropolymer-based drug-eluting stent in femoropopliteal practice: predictors of restenosis and aneurysmal degeneration. JACC Cardiovasc Interv.

[14] Soga Y., Takahara M., Iida O. (Published online November 21, 2024). Clinical outcomes following low-dose second-generation "ranger" drug-coated balloon angioplasty for femoropopliteal artery disease. J Endovasc Ther.

[15] von Allmen R.S., Weiss S., Tevaearai H.T. (2015). Completeness of follow-up determines validity of study findings: results of a prospective repeated measures cohort study. PLoS One.

[16] Rubin D.B. (1987).

[17] Wasmuth S., Baumgartner I., Do D.D. (2010). Renal insufficiency is independently associated with a distal distribution pattern of symptomatic lower-limb atherosclerosis. Eur J Vasc Endovasc Surg.

[18] Kobayashi T., Hamamoto M., Okazaki T., Hasegawa M., Takahashi S. (2021). Does the Global Limb Anatomic Staging System inframalleolar modifier influence long term outcomes of chronic limb threatening ischaemia after distal bypass?. Eur J Vasc Endovasc Surg.

[19] Toyoshima T., Iida O., Hata Y. (2023). Effects of infra-malleolar status according to Global Limb Anatomic Staging System on clinical outcomes in patients with chronic limb-threatening ischemia. Angiology.

[20] Hata Y., Iida O., Masuda M. (2024). Incidence of angiographic deterioration following inframalleolar angioplasty and its impact on outcomes in patients with chronic limb-threatening ischemia requiring repeat intervention. Circ Rep.

[21] Mentias A., Qazi A., McCoy K. (2020). Trends in hospitalization, management, and clinical outcomes among veterans with critical limb ischemia. Circ Cardiovasc Interv.

[22] Agarwal S., Sud K., Shishehbor M.H. (2016). Nationwide trends of hospital admission and outcomes among critical limb ischemia patients: from 2003-2011. J Am Coll Cardiol.

[23] Coudene A., Lapébie F.X., Desormais I. (2021). Evolution of major amputation risk in patients hospitalized in France for critical limb ischemia: the COPART registry. Angiology.

[24] Ferraresi R., Ucci A., Pizzuto A. (2021). a novel scoring system for small artery disease and medial arterial calcification is strongly associated with major adverse limb events in patients with chronic limb-threatening ischemia. J Endovasc Ther.

[25] Gray W.A., Keirse K., Soga Y. (2018). A polymer-coated, paclitaxel-eluting stent (Eluvia) versus a polymer-free, paclitaxel-coated stent (Zilver PTX) for endovascular femoropopliteal intervention (IMPERIAL): a randomised, non-inferiority trial. Lancet.

[26] Laird J.R., Schneider P.A., Tepe G. (2015). Durability of treatment effect using a drug-coated balloon for femoropopliteal lesions: 24-month results of IN.PACT SFA. J Am Coll Cardiol.

[27] Lammer J., Zeller T., Hausegger K.A. (2013). Heparin-bonded covered stents versus bare-metal stents for complex femoropopliteal artery lesions: the randomized VIASTAR trial (Viabahn endoprosthesis with PROPATEN bioactive surface [VIA] versus bare nitinol stent in the treatment of long lesions in superficial femoral artery occlusive disease). J Am Coll Cardiol.

[28] Tomoi Y., Soga Y., Imada K. (Published online January 9, 2024). Impact of a less than 50% residual stenosis following vessel preparation in femoropopliteal drug-coated balloon angioplasty. J Endovasc Ther.

[29] Soga Y., Takahara M., Yamauchi Y. (2025). Efficacy of Rheocarna®, a novel apheresis device, in patients with no- or poor-option chronic limb-threatening ischemia. Circ J.

[30] Varcoe R.L., DeRubertis B.G., Kolluri R. (2024). Drug-eluting resorbable scaffold versus angioplasty for infrapopliteal artery disease. N Engl J Med.

