# Supplementary material for: Trends in Characteristics and Treatment of Patients With Chronic Limb-Threatening Ischemia Undergoing Endovascular Therapy
Source: JACC Asia. 2025 Aug 21;5(10):1317–26. doi: 10.1016/j.jacasi.2025.06.019 (PMC12790078; doi:10.1016/j.jacasi.2025.06.019)

**Supplemental Material**

**Supplemental Table 1.** The STROBE checklist of items included in reports of observational studies

|  | Item No. | Recommendation | Page  No. | Relevant text from manuscript |
| --- | --- | --- | --- | --- |
| **Title and abstract** | 1 | (*a*) Indicate the study’s design with a commonly used term in the title or the abstract | 3 | This multicenter retrospective study included 2,085 patients with CLTI who underwent endovascular therapy between April 2010 and March 2023. |
|  |  | (*b*) Provide in the abstract an informative and balanced summary of what was done and what was found | 3 |  |
| Introduction | | | |  |
| Background/rationale | 2 | Explain the scientific background and rationale for the investigation being reported | 6 | However, due to the accumulation of technical experience and the development of endovascular devices, a less invasive endovascular approach has gained increasing acceptance among real-world patients with CLTI and complex comorbidities compared to surgical interventions. |
| Objectives | 3 | State specific objectives, including any prespecified hypotheses | 7 | Therefore, this study aimed to investigate the trends in characteristics and treatment of patients with CLTI undergoing EVT. |
| Methods | | | |  |
| Study design | 4 | Present key elements of study design early in the paper | 7 | This retrospective, multicenter study used the SAtellite database |
| Setting | 5 | Describe the setting, locations, and relevant dates, including periods of recruitment, exposure, follow-up, and data collection | 7-9 |  |
| Participants | 6 | (*a*) *Cohort study*—Give the eligibility criteria, and the sources and methods of selection of participants. Describe methods of follow-up  *Case-control study*—Give the eligibility criteria, and the sources and methods of case ascertainment and control selection. Give the rationale for the choice of cases and controls  *Cross-sectional study*—Give the eligibility criteria, and the sources and methods of selection of participants | 7-9 |  |
|  |  | (*b*) *Cohort study*—For matched studies, give matching criteria and number of exposed and unexposed  *Case-control study*—For matched studies, give matching criteria and the number of controls per case | NA |  |
| Variables | 7 | Clearly define all outcomes, exposures, predictors, potential confounders, and effect modifiers. Give diagnostic criteria, if applicable | 10 |  |
| Data sources/ measurement | 8* | For each variable of interest, give sources of data and details of methods of assessment (measurement). Describe comparability of assessment methods if there is more than one group | 7 |  |
| Bias | 9 | Describe any efforts to address potential sources of bias | 10-11 |  |
| Study size | 10 | Explain how the study size was arrived at | 7 | We excluded 77 patients with missing data, and the remaining 2085 patients were included in the present study. |

Continued on next page

| Quantitative variables | 11 | Explain how quantitative variables were handled in the analyses. If applicable, describe which groupings were chosen and why | 7 | The clinical characteristics and outcomes were compared among four groups classified based on the quartiles of the treatment periods (Q1–4) |
| --- | --- | --- | --- | --- |
| Statistical methods | 12 | (*a*) Describe all statistical methods, including those used to control for confounding | 10-11 |  |
|  |  | (*b*) Describe any methods used to examine subgroups and interactions | 10-11 |  |
|  |  | (*c*) Explain how missing data were addressed | 10-11 |  |
|  |  | (*d*) *Cohort study*—If applicable, explain how loss to follow-up was addressed  *Case-control study*—If applicable, explain how matching of cases and controls was addressed  *Cross-sectional study*—If applicable, describe analytical methods taking account of sampling strategy | 10-11 | We evaluated the follow-up index of each patient group to variate to avoid attrition bias. |
|  |  | (*e*) Describe any sensitivity analyses | 10 | Additionally, as a sensitivity analysis for reintervention, we performed a Fine–Gray test treating death or major amputation as competing events. |
| Results | | | | |
| Participants | 13* | (a) Report numbers of individuals at each stage of study—eg numbers potentially eligible, examined for eligibility, confirmed eligible, included in the study, completing follow-up, and analyzed | 7 |  |
|  |  | (b) Give reasons for non-participation at each stage | 7 |  |
|  |  | (c) Consider use of a flow diagram | NA |  |
| Descriptive data | 14* | (a) Give characteristics of study participants (eg demographic, clinical, social) and information on exposures and potential confounders | Table 1 |  |
|  |  | (b) Indicate number of participants with missing data for each variable of interest | Supplemental table |  |
|  |  | (c) *Cohort study*—Summarize follow-up time (eg, average and total amount) | Table 1 |  |
| Outcome data | 15* | *Cohort study*—Report numbers of outcome events or summary measures over time | 12 |  |
|  |  | *Case-control study—*Report numbers in each exposure category, or summary measures of exposure |  |  |
|  |  | *Cross-sectional study—*Report numbers of outcome events or summary measures |  |  |
| Main results | 16 | (*a*) Give unadjusted estimates and, if applicable, confounder-adjusted estimates and their precision (eg, 95% confidence interval). Make clear which confounders were adjusted for and why they were included | 12 |  |
|  |  | (*b*) Report category boundaries when continuous variables were categorized | NA |  |
|  |  | (*c*) If relevant, consider translating estimates of relative risk into absolute risk for a meaningful time period | NA |  |

Continued on next page

| Other analyses | 17 | Report other analyses done—eg analyses of subgroups and interactions, and sensitivity analyses | 12 | Fine–Gray test treating death or major amputation as competing events demonstrated consistent result that use of antirestenotic treatment was significantly associated with reintervention (HR 0.80 [95% CI 0.73–0.87, p = 0.028]). |
| --- | --- | --- | --- | --- |
| Discussion | | | | |
| Key results | 18 | Summarize key results with reference to study objectives | 13 | This study revealed that, over time, patient risk increased, wound severity decreased, and the anatomical severity of IM lesions increased. Additionally, the mortality or major amputation rates did not significantly differ across the treatment period quartiles, whereas the reintervention rates decreased (Central Illustration). |
| Limitations | 19 | Discuss limitations of the study, taking into account sources of potential bias or imprecision. Discuss both direction and magnitude of any potential bias | 16 |  |
| Interpretation | 20 | Give a cautious overall interpretation of results considering objectives, limitations, multiplicity of analyses, results from similar studies, and other relevant evidence | 13-16 |  |
| Generalizability | 21 | Discuss the generalizability (external validity) of the study results | 16 | … the limited generalizability of our results stemming from four centers in a specific geographic region in Japan. |
| Other information | |  | | |
| Funding | 22 | Give the source of funding and the role of the funders for the present study and, if applicable, for the original study on which the present article is based | 2 | Funding: This research received no specific grants from any funding agency in the public, commercial, or not-for-profit sectors. |

*Give information separately for cases and controls in case-control studies and, if applicable, for exposed and unexposed groups in cohort and cross-sectional studies.

**Note:** An Explanation and Elaboration article discusses each checklist item and gives methodological background and published examples of transparent reporting. The STROBE checklist is best used in conjunction with this article (freely available on the Web sites of PLoS Medicine at http://www.plosmedicine.org/, Annals of Internal Medicine at http://www.annals.org/, and Epidemiology at http://www.epidem.com/). Information on the STROBE Initiative is available at www.strobe-statement.org.

**Supplemental Table 2.** Baseline characteristics of patients regarding to data missingness

|  | Missing (-) | Missing (+) | p value |
| --- | --- | --- | --- |
| No. of patients | 876 | 1209 |  |
| Patient characteristic |  |  |  |
| Male | 331 (37.8) | 483 (40.0) | 0.34 |
| Age, years | 76 (69, 81) | 76 (70, 83) | 0.055 |
| Body mass index, kg/m² | 21.3 (19.0, 23.6) | 21.0 (18.6, 23.7) | 0.30 |
| Non-ambulatory status | 484 (55.3) | 687 (56.8) | 0.50 |
| Hypertension | 289 (33.0) | 389 (32.2) | 0.71 |
| Diabetes mellitus | 302 (34.5) | 501 (41.4) | 0.001 |
| Dyslipidemia | 491 (56.1) | 711 (58.8) | 0.21 |
| Current smoking | 737 (84.1) | 978 (80.9) | 0.018 |
| Hemodialysis | 410 (46.8) | 666 (55.1) | <0.001 |
| Coronary artery disease | 423 (48.3) | 608 (50.5) | 0.33 |
| Congestive heart failure | 779 (88.9) | 1058 (87.5) | 0.34 |
| Left ventricular ejection fraction, % | 64 (55, 69) | 64 (55, 70) | 0.35 |
| Serum albumin, g/dL | 76.0 (69.0, 81.3) | 3.3 (2.9, 3.7) | 0.48 |
| Medications |  |  |  |
| Antiplatelet agent | 106 (12.1) | 147 (12.2) | 1.00 |
| Warfarin | 725 (82.8) | 1049 (86.8) | 0.013 |
| DOAC | 831 (94.9) | 1094 (90.5) | <0.001 |
| Statin | 575 (65.6) | 803 (66.4) | 0.74 |
| ACE inhibitor / ARB | 604 (68.9) | 810 (67.1) | 0.39 |
| Beta-blocker | 596 (68.0) | 846 (70.1) | 0.34 |
| GDMT | 719 (82.1) | 972 (80.5) | 0.40 |
| Patient risk |  |  |  |
| PREVENT III score |  |  | 0.081 |
| Low risk | 99 (11.3) | 216 (17.9) | <0.001 |
| Medium risk | 341 (38.9) | 513 (42.6) |  |
| High risk | 436 (49.8) | 476 (39.5) |  |
| SPINACH score |  |  | <0.001 |
| Average risk | 674 (76.9) | 919 (76.0) |  |
| High risk | 202 (23.1) | 250 (20.7) |  |
| Missing data | 0 (0.0) | 40 (3.3) |  |
| Limb characteristic |  |  |  |
| Ankle-brachial index | 0.66 (0.53, 0.78) | 0.61 (0.46, 0.75) | <0.001 |
| Skin perfusion pressure, mmHg | 25 (16, 36) | 23 (15, 39) | 0.60 |
| Rutherford classification |  |  | <0.001 |
| 4 (only rest pain) | 65 (7.4) | 221 (18.3) |  |
| 5 (minor tissue loss) | 617 (70.4) | 716 (59.2) |  |
| 6 (major tissue loss) | 194 (22.1) | 272 (22.5) |  |
| Wound grade according to WIfI classification |  |  | < 0.001 |
| 0 | 65 (7.4) | 221 (18.7) |  |
| 1 | 401 (45.8) | 482 (40.8) |  |
| 2 | 269 (30.7) | 288 (24.4) |  |
| 3 | 141 (16.1) | 191 (16.2) |  |
| Ischemia grade according to WIfI classification |  |  | 0.11 |
| 1 | 45 (5.1) | 85 (7.3) |  |
| 2 | 194 (22.1) | 248 (21.2) |  |
| 3 | 199 (22.7) | 293 (25.1) |  |
| Foot infection grade according to WIfI classification |  |  | < 0.001 |
| 0 | 369 (42.1) | 611 (51.7) |  |
| 1 | 233 (26.6) | 253 (21.4) |  |
| 2 | 239 (27.3) | 256 (21.7) |  |
| 3 | 35 (4.0) | 61 (5.2) |  |
| WIfI clinical stage |  |  | < 0.001 |
| Stage 1 (very low risk) | 89 (10.2) | 141 (11.7) |  |
| Stage 2 (low risk) | 140 (16.0) | 288 (23.9) |  |
| Stage 3 (moderate risk) | 259 (29.6) | 309 (25.7) |  |
| Stage 4 (high risk) | 388 (44.3) | 409 (34.0) |  |
| Arterial lesion characteristic |  |  |  |
| GLASS FP grade |  |  | < 0.001 |
| 0 | 352 (40.2) | 338 (29.9) |  |
| 1 | 120 (13.7) | 138 (12.2) |  |
| 2 | 128 (14.6) | 133 (11.8) |  |
| 3 | 122 (13.9) | 167 (14.8) |  |
| 4 | 154 (17.6) | 354 (31.3) |  |
| GLASS IP grade |  |  | < 0.001 |
| 0 | 44 (5.0) | 111 (10.2) |  |
| 1 | 81 (9.2) | 70 (6.5) |  |
| 2 | 117 (13.4) | 140 (12.9) |  |
| 3 | 131 (15.0) | 184 (17.0) |  |
| 4 | 503 (57.4) | 579 (53.4) |  |
| Missing data | 57 (10.9) | 32 (6.1) |  |
| GLASS IM grade |  |  | 0.51 |
| P0 | 270 (30.8) | 262 (28.4) |  |
| P1 | 368 (42.0) | 393 (42.7) |  |
| P2 | 238 (27.2) | 266 (28.9) |  |
| GLASS stage |  |  | <0.001 |
| Stage I | 132 (15.1) | 97 (8.2) |  |
| Stage II | 149 (17.0) | 184 (15.6) |  |
| Stage III | 595 (67.9) | 809 (68.5) |  |
| Lesion distribution |  |  |  |
| FP | 352 (40.2) | 338 (29.9) | <0.001 |
| IP | 44 (5.0) | 111 (10.2) | <0.001 |
| IM | 270 (30.8) | 262 (28.4) | 0.28 |
| Treatment modality |  |  | 0.39 |
| Ballon-based treatment | 550 (62.8) | 730 (60.4) |  |
| Stent-based treatment | 140 (16.0) | 192 (15.9) |  |
| Antirestenotic treatment | 186 (21.2) | 287 (23.7) |  |
| Preferred revascularization strategy by the GVG |  |  | <0.001 |
| EVT preferred or indeterminate | 557 (63.6) | 674 (55.7) |  |
| BSX preferred | 319 (36.4) | 349 (28.9) |  |

Data are presented as n (%) or median (interquartile range). Missing (+) indicates cases in which at least one of the collected data elements was absent. DOAC: direct oral anticoagulant, ACE: angiotensin-converting enzyme, ARB: angiotensin receptor blocker, GDMT: Guideline-directed medical therapy (aggregate prescription of antiplatelet agents, statins, and ACE inhibitors or ARBs), WIfI: Wound, ischemia, and foot infections, GLASS: Global limb anatomical staging system, FP: femoropopliteal, IP: infra-popliteal, IM: infra-malleolar. GVG: global vascular guideline. EVT: endovascular therapy. BSX: bypass surgery. WIfI clinical stage was used to predict the 1-year amputation risk. Balloon- and stent-based treatments were defined as those using plain balloons and bare metal stents, respectively. Antirestenotic treatment was defined as treatment with drug-coated balloons, drug-eluting stents, or stent grafts. In cases where both limbs were treated, the limb with the higher WIfI stage was used for data analysis to capture the more severe presentation.

**Supplemental Table 3.** Association between baseline characteristics and data missingness

|  | Multivariate model | |
| --- | --- | --- |
|  | OR [95% CI] | p value |
| Age | 1.00 [0.99-1.01] | 0.74 |
| Non-ambulatory status | 1.05 [0.87-1.27] | 0.61 |
| Diabetes mellitus | 0.89 [0.73-1.08] | 0.23 |
| Hemodialysis | 0.81 [0.67-0.98] | 0.031 |
| Congestive heart failure | 1.15 [0.87-1.53] | 0.33 |
| Tissue loss | 0.46 [0.33-0.65] | <0.001 |
| WIfI clinical stage | 0.91 [0.82-1.01] | 0.069 |
| GLASS stage | 1.33 [1.16-1.52] | <0.001 |

Odds ratios (ORs) are presented with 95% confidence intervals (CIs).

WIfI: Wound, ischemia, and foot infections, GLASS: Global limb anatomical staging system,

**Supplemental Table 4.** Details of treatment modalities

|  | Q1 | Q2 | Q3 | Q4 | p value |
| --- | --- | --- | --- | --- | --- |
| No. of patients | 521 | 521 | 522 | 521 |  |
| Treatment modality |  |  |  |  | <0.001 |
| Balloon angioplasty | 337 (64.8) | 363 (69.7) | 308 (59.0) | 272 (52.2) |  |
| Bare metal stent | 134 (25.7) | 127 (24.4) | 42 (8.0) | 11 (2.1) |  |
| Stent graft | 0 (0) | 11 (2.1) | 23 (4.4) | 20 (3.8) |  |
| Drug-coated balloon | 0 (0) | 0 (0) | 79 (15.1) | 160 (30.7) |  |
| Drug-eluting stent | 50 (9.6) | 20 (3.8) | 57 (10.9) | 53 (10.2) |  |
| Interwoven stent | 0 (0) | 0 (0) | 13 (2.5) | 5 (1.0) |  |

Data are presented as n (%). Q1–4:1st to 4th quartiles

**Supplemental Figure 1.** Mortality rates

One-year mortality rates were 18.9% (95% CI 15.5%-22.8%), 17.8% (95% CI 14.5%-21.9%), 18.9% (95% CI 15.5%-22.9%), and 24.0% (95% CI 20.1%-28.5%) in Q1, Q2, Q3, and Q4, respectively, without significant differences (log-rank p = 0.080 and p for trend = 0.30). Dashed lines indicated the 95% CIs.

CI: confidence interval. Q1–4: 1st to 4th quartiles


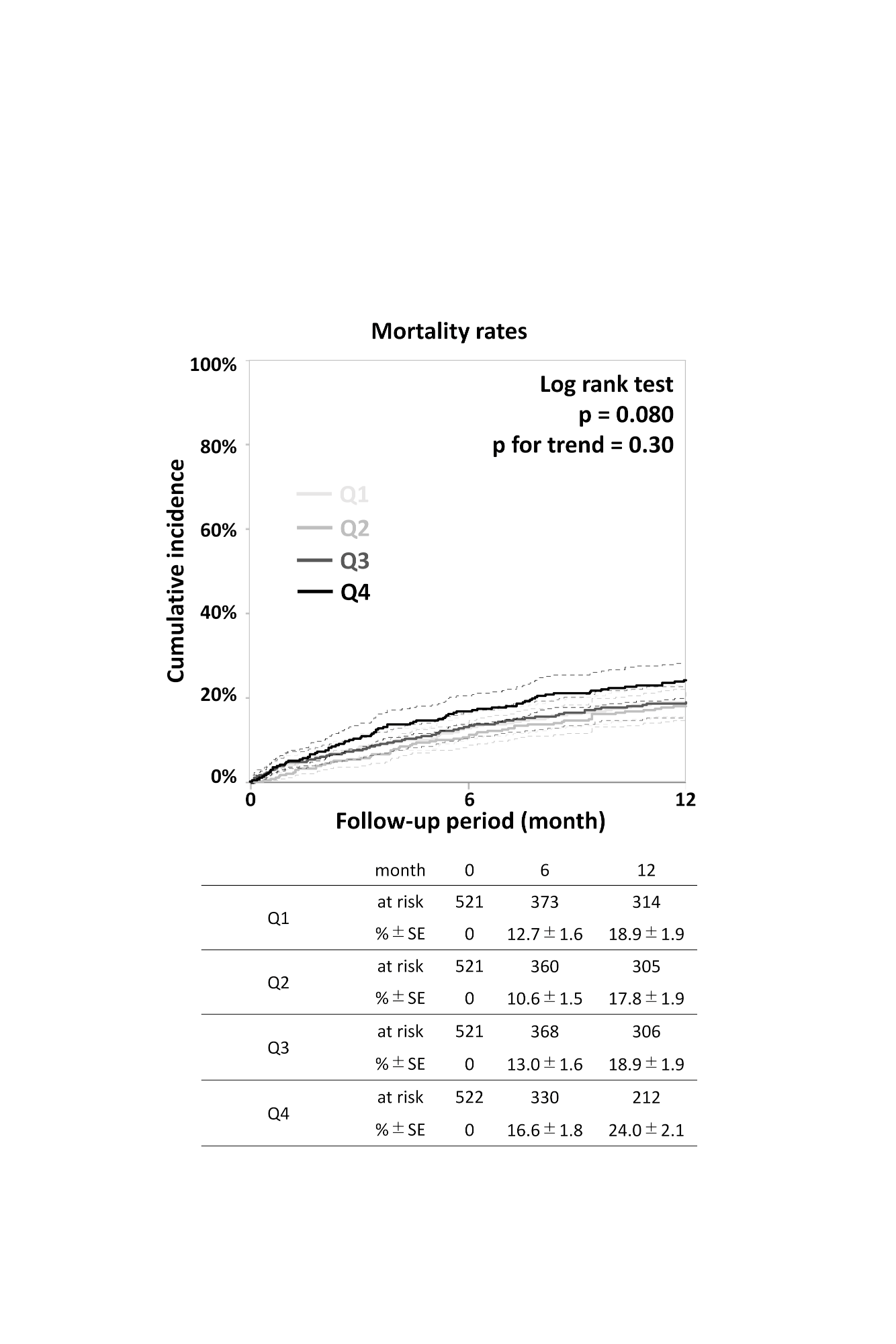


**Supplemental Figure 2**. Major amputation rates

One-year major amputation rates were 8.6% (95% CI 6.3%-11.6%), 8.8% (95% CI 6.4%-12.0%), 10.0% (95% CI 7.5%-13.3%), and 8.9% (95% CI 6.5%-12.1%) in Q1, Q2, Q3, and Q4, respectively, without significant differences (log-rank p = 0.65 and p for trend = 0.41). Dashed lines indicated the 95% CIs.

CI: confidence interval. Q1–4: 1st to 4th quartiles


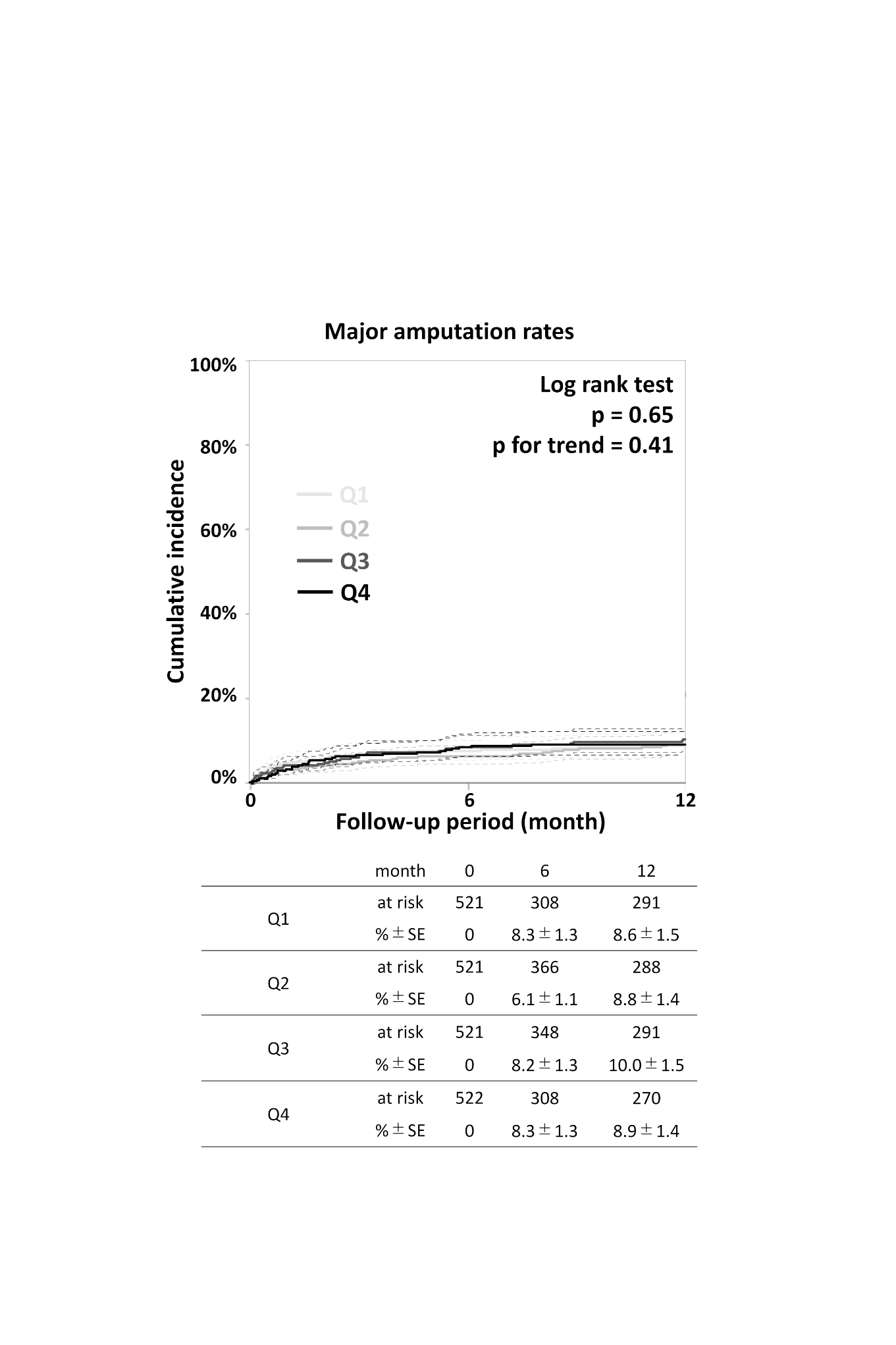

Supplement: Supplemental Tables 1-4 and Supplemental Figures 1 and 2 [file mmc1.docx]
